# Comprehensive proteomic investigation of infectious and inflammatory changes in late preterm prelabour rupture of membranes

**DOI:** 10.1038/s41598-020-74756-9

**Published:** 2020-10-19

**Authors:** Marie Vajrychová, Jaroslav Stráník, Kristýna Pimková, Malin Barman, Rudolf Kukla, Petra Zedníková, Radka Bolehovská, Lenka Plíšková, Helena Hornychová, Ctirad Andrýs, Vojtěch Tambor, Juraj Lenčo, Bo Jacobsson, Marian Kacerovský

**Affiliations:** 1 Biomedical Research Center, University Hospital Hradec Kralove, Hradec Kralove, Czech Republic; 2 Department of Obstetrics and Gynecology, University Hospital Hradec Kralove, Charles University, Faculty of Medicine in Hradec Kralove, Hradec Kralove, Czech Republic; 3 Food and Nutrition Science, Department of Biology and Biological Engineering, Chalmers University of Technology, Gothenburg, Sweden; 4 Institute of Clinical Microbiology, University Hospital Hradec Kralove, Hradec Kralove, Czech Republic; 5 Department of Biological and Biochemical Sciences, Faculty of Chemical Technology, University of Pardubice, Pardubice, Czech Republic; 6 Institute of Clinical Biochemistry and Diagnostics, University Hospital Hradec Kralove, Hradec Kralove, Czech Republic; 7 Fingerland’s Department of Pathology, University Hospital in Hradec Kralove, Charles University, Faculty of Medicine Hradec Kralove, Hradec Kralove, Czech Republic; 8 Institute of Clinical Immunology and Allergology, University Hospital Hradec Kralove, Hradec Kralove, Czech Republic; 9 Department of Analytical Chemistry, Faculty of Pharmacy in Hradec Kralove, Charles University, Hradec Kralove, Czech Republic; 10 Department of Obstetrics and Gynecology, Institute of Clinical Science, Sahlgrenska Academy, University of Gothenburg, Gothenburg, Sweden; 11 Department of Genetics and Bioinformatics, Domain of Health Data and Digitalization, Institute of Public Health, Oslo, Norway; 12 Present Address: BIOCEV, First Faculty of Medicine, Charles University, Prague, Czech Republic; 13 Present Address: Department of Metabolomics, Institute of Physiology, Czech Academy of Sciences, Prague, Czech Republic

**Keywords:** Inflammation, Infectious-disease diagnostics, Infection, Inflammation, Preterm birth

## Abstract

Preterm prelabour rupture of membranes beyond the 34th week of gestation (late PPROM) is frequently associated with the risk of the microbial invasion of the amniotic fluid (MIAC) and histological chorioamnionitis (HCA). Hence, we employed a Tandem Mass Tag-based approach to uncover amniotic fluid proteome response to the presence of MIAC and HCA in late PPROM. Protein dysregulation was associated with only five cases in the group of 15 women with confirmed MIAC and HCA. Altogether, 138 amniotic fluid proteins were changed in these five cases exclusively. These proteins were particularly associated with excessive neutrophil responses to infection, such as neutrophil degranulation and extracellular trap formation. We believe that the quantification of these proteins in amniotic fluid may assist in revealing women with the highest risk of excessive inflammatory response in late PPROM.

## Introduction

Preterm prelabour rupture of membranes (PPROM) is defined as leakage of amniotic fluid prior to 37 weeks of gestation and the leakage of amniotic fluid happening at least two hours prior to the onset of regular uterine contractions^1^. Late PPROM, occurring between 34 and 37 weeks of gestation, represents more than 50% of cases^2^. Despite being considered as “near-term,” late PPROM is associated with significant short-term neonatal morbidity, mainly respiratory distress syndrome and a higher risk of early-onset neonatal sepsis^3^. Concerns about the long-term consequences have also been raised because children born late preterm are at a higher risk of adverse developmental outcomes up to 7 years of age^4,5^.

Women with late PPROM can be managed actively with an early planned delivery or expectantly through an attempt to prolong the pregnancy. The aim of the active approach is to avoid prolonged fetal exposure to a potential intra-amniotic infection. On the other hand, expectant management may reduce the incidence of neonatal morbidity (especially respiratory distress syndrome) as the neonates are more matured^6^. A recent meta-analysis did not reveal evidence of a higher incidence of early-onset neonatal sepsis in the group with expectant management^7^. In addition, neonates in the active-management group were more prone to develop the respiratory distress syndrome^7^. The authors therefore suggest that expectant management is an acceptable alternative to immediate delivery even in the near-term pregnancy.

Nonetheless, expectant management for all women is still controversial and most national guidelines recommend planned delivery for PPROM shortly after 34 weeks of gestation^8,9^. The main rationale for the guidelines is the possible presence of subclinical complications such as microbial invasion of the amniotic cavity (MIAC) and intra-amniotic inflammation such as acute histological chorioamnionitis (HCA). These complications are related to the highest incidence of fetal inflammatory response syndrome and short-term neonatal morbidity^10–12^. Moreover, MIAC and HCA are frequently asymptomatic and therefore difficult to being detected. The current dilemma with the delivery timing suggests that a universal approach may not offer an apparent benefit in all cases. Early identification of cases with intra-amniotic and intrauterine complications and personalized clinical management might improve the outcomes of late PPROM pregnancies.

Our group has previously shown that the presence of both MIAC and HCA in women with late PPROM was associated with an increase of amniotic fluid interleukin-6 (IL-6) in comparison with women with either HCA alone, MIAC alone or with absence of both conditions^13^. However, little is known about the intra-amniotic environment in terms of other proteins, when MIAC and HCA are present in late PPROM. Moreover, the concentration of amniotic fluid IL-6 in the presence of MIAC and HCA may vary from hundreds to hundreds of thousands pg/mL^14^. Thus, the heterogeneity of individual cases may hamper proper assessment of how severe the intra-amniotic inflammation is.

**Figure 1:**
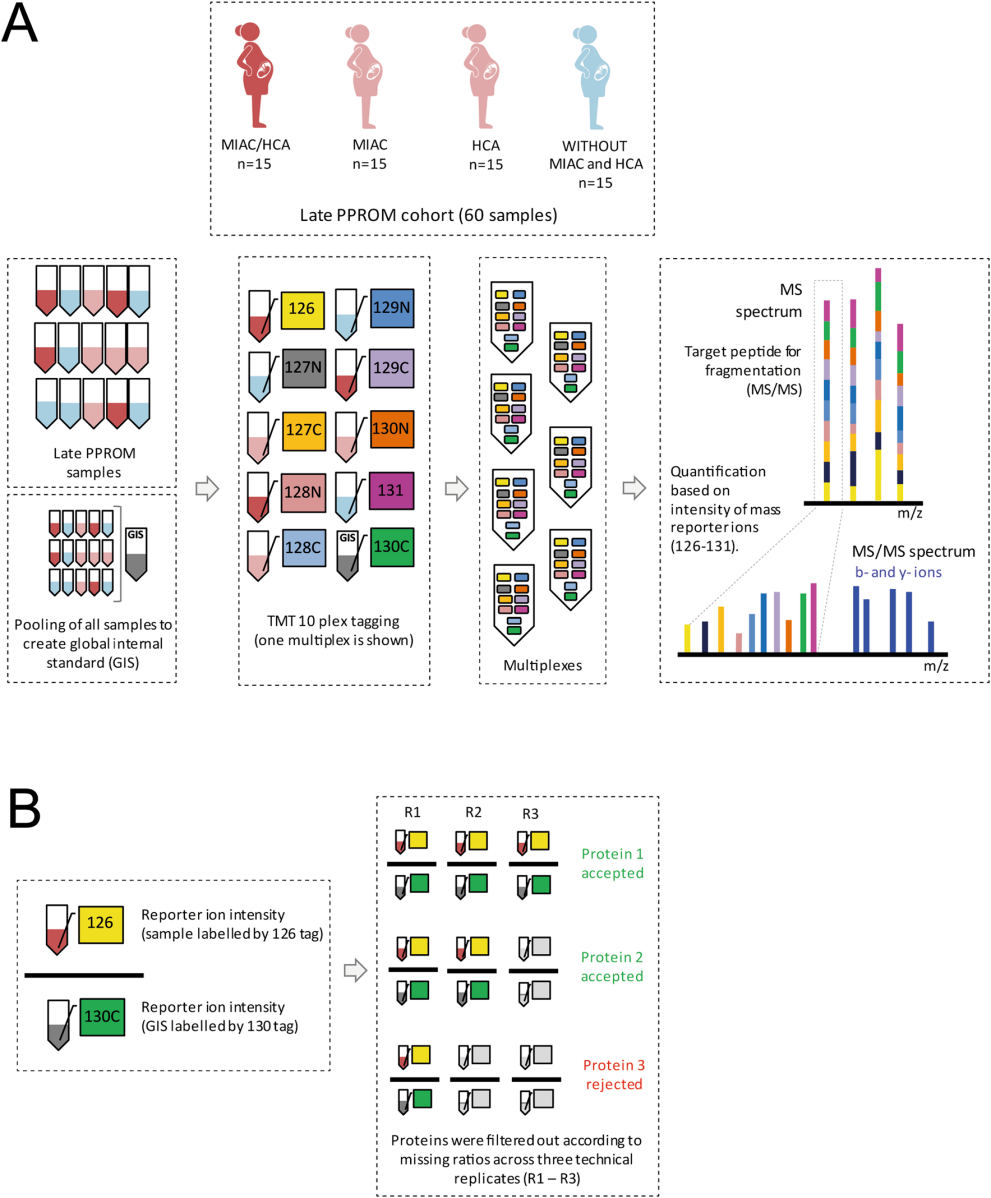
(**A**) Multiplex labeling strategy. Four subgroups of amniotic fluid samples were collected: MIAC and HCA both present, MIAC present alone, HCA present alone, without MIAC and HCA. An aliquot of each sample was taken, and the aliquots were pooled to prepare a global internal standard (GIS). After protein digestion, amniotic fluid samples (n = 60) and GIS were labeled with ten isobaric mass tags (126–131). Nine differently labeled samples and one labeled GIS aliquot formed one multiplex. In total, seven multiplexes were analyzed by different LC–MS/MS experiments. In MS spectra, peptides derivatized with different isotopic tags are present as a single peak with the same value of *m/z* and retention time. The fragmentation of peptide precursors separates the reporter ions and fragment ions. The difference in peptide level among individual samples is assessed according to the relative intensity of reporter ions in MS/MS spectra. The fragment ions, mostly b- and y-, are used for peptide identification. The schematic illustration of late PPROM cohort was created with BioRender.com. (**B**) Normalization of individual samples to the global internal standard and removal of proteins with a high number of missing values. The intensities of mass reporter ions were divided by the mass reporter intensity of GIS quantified in the same MS/MS spectrum and the ratios were compared across defined late PPROM subgroups. Proteins with more than one missing value from three technical replicates in more than eight samples were excluded from the final dataset.

Despite these facts, a complex proteomic study focused solely on late PPROM is lacking. Liquid chromatography separation coupled to tandem mass spectrometry (LC–MS/MS) enables identification of thousands of proteins in different kinds of biological samples^15^. One of the main and widely used approaches of protein quantification is multiplexed isobaric tagging allowing simultaneous protein quantification in multiple samples. In this approach, peptides processed from previous protein digestion are labeled with a chemical tag (reporter) in each sample. Differently-labeled samples are then combined before LC–MS/MS analysis. The fragmentation of peptide precursors separates the reporter. Subsequently, the difference in peptide level among samples is assessed according to the relative abundance of reporters in the fragmentation spectra^16^. Finally, protein quantification is based on the relative quantification of tagged peptides, unique to identified proteins (Fig. 1A).

The aim of this study was to take advantage of the modern methodology to assess how the amniotic fluid proteome responds to the presence of MIAC and/or HCA in women with late PPROM. We believed that these findings might improve our understanding of the molecular processes taking place due to MIAC and HCA in late PPROM pregnancies, and hence facilitate personalized clinical management.

## Methods

### Reagents and Material

Unless otherwise indicated, chemicals and reagents were purchased from Sigma-Aldrich (St. Louis, MO, USA) in the highest available grade.

### Ethical approval

This study was approved by the Institutional Review Board—Ethics Committee, University Hospital Hradec Kralove (March 19, 2008, No. 200804 SO1P; and July 2014, No. 201407 S14P). All research was performed in accordance with relevant guidelines and regulations. Informed consent was obtained from all participants.

### Design of late PPROM cohort

The late PPROM cohort was completed considering the following criteria as described previously^17^. A total of 60 samples of amniotic fluid were collected by transabdominal amniocentesis. Women older than 18 years with a singleton pregnancy, with gestational age between 34 and 37th week, and admitted with a diagnosis of PPROM were included. The diagnosis of PPROM was conditioned by pooling of amniotic fluid in the vagina and detection of insulin-like growth factor-binding protein 1 (ACTIM PROM test; MedixBiochemica, Kauniainen, Finland) in the vaginal fluid. Pregnancy complications such as intrauterine growth restriction, structural malformation or chromosomal abnormality of the fetus, maternal hypertension, diabetes mellitus, pre-eclampsia, thyroid disease, vaginal bleeding, and fetal hypoxia were exclusion criteria. All clinical characteristics are listed in Table 1. Potential differences among the subgroups of late PPROM were tested by nonparametric Kruskal–Wallis test for continuous variables and are presented in Table 1 as median with interquartile range. Categorical variables were compared by the χ^2^ test and are presented as numbers (%).

**Table 1:** Clinical characteristics of the late PPROM cohort.

	MIAC and HCA (n = 15)	MIAC alone (n = 15)	HCA alone (n = 15)	without MIAC and HCA (n = 15)	*p*
Maternal age	31 (26–37)	31 (25–33)	33 (28–36)	33 (31–36)	0.13
Primiparous	7 (47%)	7 (47%)	10 (67%)	4 (27%)	0.19
Prepregnancy BMI [kg/m^2^]	22.5 (20.5–24.8)	23.4 (20.2–25.5)	25.0 (22.7–28.9)	24.8 (21.2–27.9)	0.15
Smoking	3 (20%)	4 (27%)	1 (7%)	0 (0%)	0.12
Gestational age at admission [weeks]	35 + 0 (34 + 0–35 + 5)	35 + 5 (34 + 3–36 + 2)	35 + 1(34 + 6–36 + 0)	35 + 5 (35 + 0–36 + 4)	0.46
Gestational age at delivery [weeks]	35 + 1 (34 + 4–35 + 5)	35 + 5 (34 + 4–36 + 3)	35 + 6 (35 + 0–36 + 0)	35 + 6 (35 + 0–36 + 4)	0.44
Latency from PPROM to amniocentesis [h]	7 (4–11)*	4 (3–7)	6 (3–8)	3 (2–6)*	**0.04**
Latency from amniocentesis to delivery [h]	10 (4–28)	6 (3–21)	18 (3–40)	9 (3–14)	0.40
Latency from PPROM to delivery [h]	24 (11–31)	13 (6–28)	25 (12–48)	12 (9–20)	0.15
CRP levels at admission [mg/L]	6.0 (3.2–22.2)	5.0 (3.7–10.0)	6.8 (4.2–10.9)	4.6 (1.7–10.2)	0.49
WBC count at admission [× 10^9^ L]	13.7 (10.8–14.4)	11.5 (9.6–13.2)	10.6 (9.2–11.4)	11.8 (9.1–13.4)	0.09
Administration of antibiotics	15 (100%)	13 (87%)	13 (87%)	15 (100%)	0.23
Administration of corticosteroids	5 (33%)	3 (20%)	2 (13%)	2 (13%)	0.48
Spontaneous vaginal delivery	12 (80%)	14 (93%)	12 (80%)	10 (67%)	0.34
Forceps delivery	0 (0%)	0 (0%)	0 (0%)	1 (7%)	0.38
Cesarean delivery	3 (20%)	1 (7%)	3 (20%)	4 (27%)	0.55
Birth weight [g]	2750 (2300–2900)	2310 (1940–2860)	2590 (2300–2950)	2610 (2300–2980)	0.35
Apgar score in 5 min	10 (9–10)	10 (9–10)	10 (9–10)	10 (9–10)	0.69
Apgar score in 10 min	10 (10–10)	10 (10–10)	10 (10–10)	10 (10–10)	0.87
Funisitis	9 (60%)	0 (0%)	5 (30%)	0 (0%)	** < 0.001**

Clinical characteristics of the women with PPROM before 37th week of gestation stratified according to the presence of MIAC and/or HCA. Continuous variables were compared using a nonparametric Kruskal–Wallis test. Categorical variables were compared using the χ^2^ test. Continuous variables are presented as median with interquartile range (IQR) and categorical as number (%). The statistically significant results are marked in bold.

Abbreviations: BMI—Body Mass Index, PPROM—preterm prelabor rupture of membranes, MIAC—microbial invasion of the amniotic cavity, HCA—histological chorioamnionitis, CRP—C-reactive protein, WBC—white blood cells.

### Diagnosis of MIAC and HCA

MIAC was established based on a qPCR detection of *Ureaplasma* spp., *Mycoplasma hominis*, *Chlamydia trachomatis,* and positivity for bacterial DNA identified by PCR targeting 16S rRNA and analyzed by sequencing, and aerobic/anaerobic culture of amniotic fluid. Detection of DNA of *Ureaplasma* spp.*, M. hominis, C. trachomatis* and other bacteria and aerobic and anaerobic cultures of amniotic fluid was carried out as in previously published work^18^. HCA was diagnosed on the basis of the degree of neutrophil infiltration that was inspected separately in the amnion, chorion-decidua, chorionic plate, and umbilical cord. The degree of infiltration was assessed according to the criteria described by Salafia^19^. A diagnosis of HCA was conditioned by the grades of infiltration 3–4 for the chorion-decidua, 3–4 for the chorionic plate, 1–4 for the umbilical cord, and/or 1–4 for the amnion. Samples were divided according to the MIAC and HCA status and were distributed into four subgroups: subgroup with the presence of both MIAC and HCA, subgroup with MIAC alone, subgroup with HCA alone, and subgroup with absence of both MIAC and HCA (Table 1).

### Amniotic fluid sample preparation

Amniotic fluid samples were prepared according to the workflow successfully applied in our previous reports^17,20^. Briefly, ultrasound-guided transabdominal amniocentesis was carried out upon admission before the administration of corticosteroids, antibiotics (penicillin G, clindamycin, azithromycin, or clarithromycin), or tocolytics. Up to five mL of amniotic fluid was supplemented with a protease inhibitor cocktail (Complete Mini, EDTA free, Roche Diagnostics, Basel, Switzerland). The treated samples were then centrifuged at 300 × g for 15 min and passed through a 0.22 μm syringe filter (TPP, Trasadingen, Switzerland) to remove cells and debris. The total protein concentration was determined by Bicinchoninic acid assay and ballast proteins were removed as described in the Supplementary Document.

### Protein digestion

Enzymatic digestion of proteins was processed in 200 mM triethylammonium bicarbonate buffer, pH 8.5 (TEAB, Thermo Scientific, Rockford, IL, USA) with 1% of sodium deoxycholate. Disulfide bonds were reduced with 5 mM tris(2-carboxyethyl) phosphine hydrochloride at 60 °C for 1 hour (h), and the free thiol groups were blocked using 10 mM methyl methanethiosulfonate at room temperature for 30 min. Amniotic fluid proteins were digested with lysyl endopeptidase (Waco Pure Chemical Industries, Osaka, Japan) at 37 °C for 4 h, followed by digestion with trypsin (Promega, Madison, WI, USA) at 37 °C overnight. Both enzymes were added at a 1:50 enzyme to protein ratio (w/w).

### Tandem Mass Tag 10plex isobaric labeling and multiplex strategy

Tandem Mass Tag (TMT) 10plex isobaric label reagents (Thermo Scientific, Rockford, IL, USA) were dissolved according to the manufacturer’s instructions. All the amniotic fluid samples were evaporated to dryness and re-suspended in 8.57 µL of 100 mM TEAB, and 1.57 µL was taken from each sample and combined to prepare a global internal standard (GIS). The total volume of the GIS was split into 10 aliquots. Individually digested samples and the GIS aliquots, both at concentration 1 µg/µL, were incubated with 10 μL of the TMT reagents at room temperature for 1 h. The reaction was terminated by adding 5% hydroxylamine. Nine amniotic fluid samples and one GIS aliquot were mixed in 1:1 ratio to create seven multiplexes (Fig. 1A). To complete the seventh multiplex, the last three GIS aliquots were labeled by 126, 128C, 129C channels. However, these labeled GIS aliquots were not considered in the data evaluation. Prepared multiplexes were cleaned by peptide extraction on solid phase as described in the Supplementary Document.

### Liquid chromatography coupled to tandem mass spectrometry (LC–MS/MS)

The multiplexes were redissolved in 0.1% trifluoroacetic acid (TFA), 2% acetonitrile (AcN) to a concentration of 1 µg/µL and injected twice on an UltiMate 3000 RSLCnano System (Thermo Scientific, Bremen, Germany) in three technical replicates. The analytical system consisted of a PepMap100 C18, 3 µm, 100 Å, 75 µm × 20 mm trap column and a PepMap RSLC C18, 2 µm, 100 Å, 75 µm × 500 mm analytical column (both from Thermo Scientific, Bremen, Germany) and was previously used in our proteomic analyses^21^. The samples were loaded onto the trap column at a flow rate 8 µl/min of 0.1% TFA, 2% AcN for 3 min. Tryptic peptides were separated via a linear gradient running from 2 to 45% of 0.1% formic acid, 80% AcN at a flow rate 200 nL/min for 240 min. Eluted peptides were sprayed into a Q Exactive Plus mass spectrometer using a Nanospray Flex ion source (Thermo Scientific, Bremen, Germany) at 1.8 kV spray voltage. Positive ion full scan MS spectra were acquired in the range of *m/z* 350–1600, with 3 × 10^6^ AGC target in the Orbitrap at 70,000 resolution with a maximum ion time of 50 ms. A lock mass of *m/z* 445.12003 ([C_2_H_6_SiO]_6_) was used for internal calibration of the mass spectra. The fragmentation (MS/MS) spectra were acquired for the 15 most intense precursors. Other parameter settings were assumed from the literature^22^. Isolation window of 1.2 m*/z* and normalized collision energy of 33 was used. Each fragmentation spectrum was acquired at a resolution of 35,000, with a 2 × 10^5^ AGC target and a maximum injection time of 100 ms. The first mass was fixed to 120 m*/z*.

### Data treatment

Recorded spectra were processed and searched in MaxQuant 1.5.5.1 against a reviewed SwissProt human protein database including contaminants downloaded in 2016. Trypsin digestion specificity with up to two missed cleavages allowed was used. Cysteine thiomethylation was set as a fixed modification and oxidation of methionine was selected as a variable modification. The mass tolerance in MS and MS/MS mode was left at the default value for the initial search and 6 ppm was set as mass tolerance in MS mode for the main search. The false discovery rate for protein identification was left at the default value. Output files from MaxQuant were processed in Perseus^23^ and R^24^. Potential contaminants and proteins identified by site and by reverse sequence were removed. The reporter intensity of proteins in each sample was divided by reporter intensity of proteins in the GIS, both adjusted to correction factors. Resulting ratios were log_2_ transformed and normalized to the median. One-way analysis of variance (ANOVA) with Benjamin–Hochberg false discovery rate (FDR) correction was applied to compare normalized protein ratios in the subgroups of late PPROM. Perseus Tukey’s significant honest difference post-hoc test on the significance level of 0.05 was used on the ANOVA significant hits. Gene ontology (GO) analysis was carried out using StringApp plugin^25^ in the Cytoscape platform^26^ (enrichment *p* value < 0.01). Violin plots, principal component analysis (PCA) plot and bubble plots were designed in the R software. Bar graphs of GO terms were prepared in GraphPad Prism 6.

### Amniotic fluid IL-6 and histone H1.5 level determination by ELISA

ELISA kit for human IL-6 (R&D systems, MN, USA) and human histone H1.5 (MyBiosource, CA, USA) were used to determine IL-6 and H1.5 levels in amniotic fluid. Samples with IL-6 concentration above the top calibration point were diluted to fit the calibration range. Resulting absorbance was read at 450 nm with correction to 540 nm. Data was evaluated in GraphPad Prism 6.

## Results

### Clinical characteristics of the patient cohort

A late PPROM cohort of 60 amniotic fluid samples with the presence or absence of MIAC and/or HCA was designed (Fig. 1A) and important clinical characteristics were compared among the subgroups (Table 1). Gestational age at delivery was considered as important clinical confounder of protein level due to a substantial increase in protein turnover during pregnancy^27^. Hence, the late PPROM cohort was carefully designed to include women in similar gestational age at admission and delivery. The median time of latency between late PPROM and amniocentesis was 4 h. We found a significant difference only between the subgroup with the presence of both MIAC and HCA (median = 7 h; IQR 4–11 h) compared to the subgroup with the absence of both MIAC and HCA (median = 3 h; IQR 2–6 h; *p* = *0.04*). The median time of latency between amniocentesis and delivery was 10 h and the median time of latency between late PPROM and delivery was 16 h. There was not any significant difference in the duration between amniocentesis/late PPROM and delivery (*p* = *0.4* and *p* = *0.15,* respectively). Based on these results, we infer that the changes of protein levels were not due to differences in the gestational age.

### Tandem Mass Tag 10plex LC–MS/MS analysis and data treatment

The wide dynamic range of protein concentration represents a major obstacle in mass spectrometry-based protein analysis in human biological fluids such as plasma or amniotic fluid. Human serum albumin (HSA) is the most abundant protein in amniotic fluid and may smother information on important but far less abundant proteins^28^. The immunoaffinity depletion of abundant ballast proteins allowed us to detect a total of 1132 amniotic fluid proteins. The protein quantity was expressed as the ratio of TMT reporter ion intensity of each identified protein to the TMT reporter ion intensity of the global internal standard (GIS) in the respective multiplex (Fig. 1B). This allowed us to determine protein level changes in all 60 samples by the implementation of ten isobaric tags. Subsequent filtration of protein contaminants and proteins with a high number of missing values reduced the dataset to 816 quantified amniotic fluid proteins. Out of those, a total of 716 proteins were quantified in at least two from three technical replicates in all samples and 100 proteins were quantified in at least two technical replicates in eight samples in each late PPROM group (Fig. 1B). Thus, 72% of the identified proteins were kept for further consideration.

### Comparative analysis of amniotic fluid proteins in the presence or absence of MIAC and/or HCA

**Figure 2:**
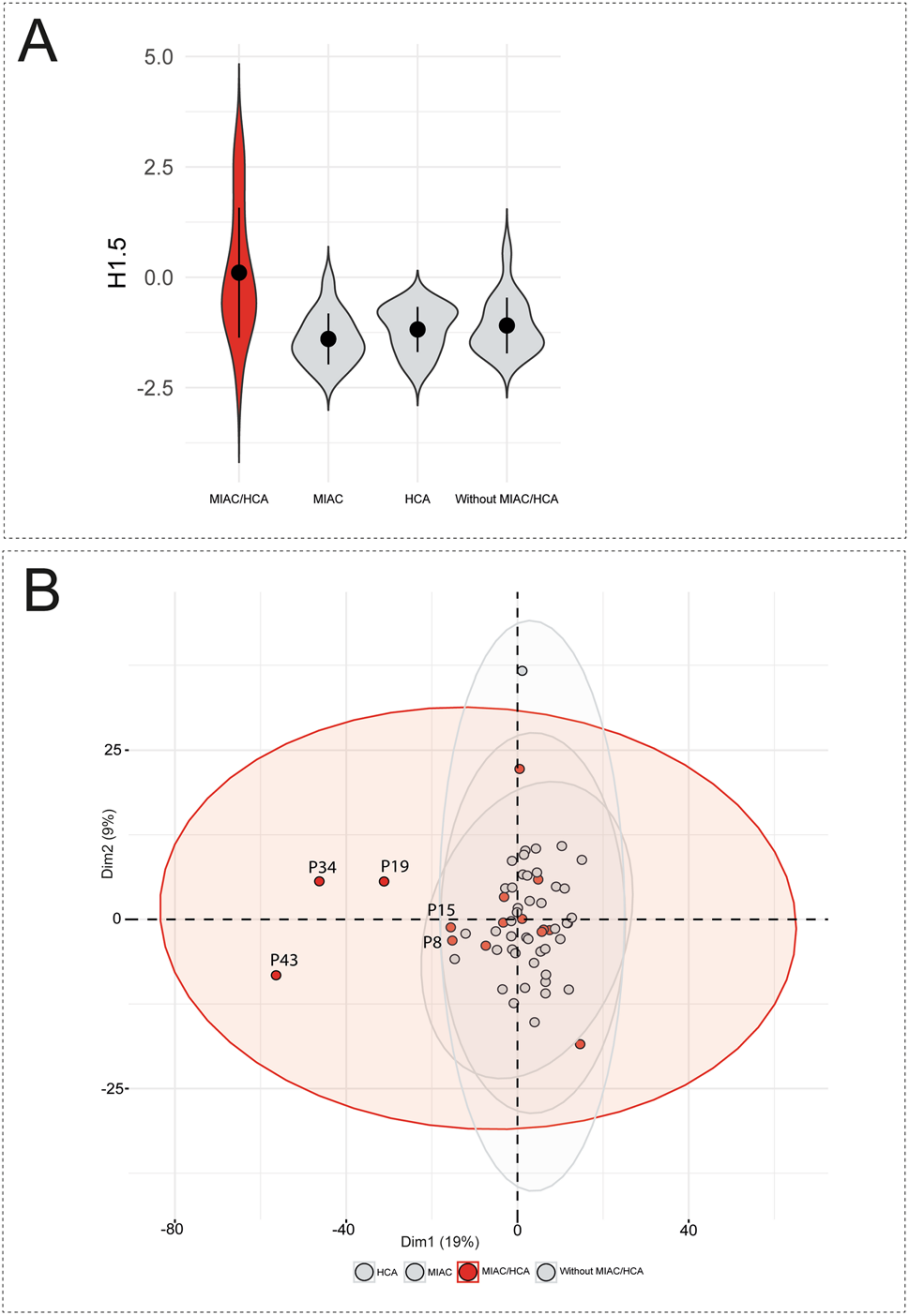
(**A**) Violin plots illustrating the heterogeneity of the subgroup with the presence of both MIAC and HCA. The construction of violin plots was based on the level of histone H1.5 in all samples. Subgroup with the presence of both MIAC and HCA is highlighted in red. Points with the error bars represent means with ± standard deviation. (**B**) PCA score plot of individual samples. Each point represents one sample. Each ellipse represents one late PPROM subgroup. Points and ellipse of the subgroup with the presence of both MIAC and HCA are highlighted in red. The position and the size of the ellipses depend on sample distribution in each group. The PCA plot shows uniqueness of the five outlying samples in comparison with the other PPROM groups regardless of MIAC and/or HCA status. The five outlying samples were labelled by identification code (P letter and number).

The initial comparison was based on determination of the protein difference in the subgroups divided according to MIAC and/or HCA (the presence of both MIAC and HCA, MIAC alone, HCA alone, and the absence of both MIAC and HCA). Firstly, the multiple comparisons of protein levels were adjusted by Benjamin-Hochberg correction with the FDR threshold 0.05. However, no significant protein changes related to MIAC and/or HCA were found. The histone H1.5 (H1.5) was found as the first protein above the FDR threshold (FDR q value = 0.0625). Thus, violin plots based on the level of H1.5 in amniotic fluid were constructed to explain the uniformity of the late PPROM subgroups despite distinct MIAC and HCA status (Fig. 2A). Although the mean of the subgroup with the presence of both MIAC and HCA was higher than the mean of other subgroups, we observed a distinctly higher variance of the subgroup with the presence of both MIAC and HCA. Subsequently, a PCA plot of all individual samples was constructed and all 816 proteins were used as variables (Fig. 2B). The PCA plot also showed a distinct distribution of the subgroup with the presence of both MIAC and HCA compared to other late PPROM subgroups. The PCA plot illustrating the distribution of all individual samples revealed a cluster of five samples from the subgroup with both MIAC and HCA. The distribution of these five samples was dissimilar compared to the distribution of the remaining samples in the PCA plot (Fig. 2B). Violin and PCA plots together illustrated substantial heterogeneity of protein response to MIAC and HCA and a unique subgroup of five women was identified. Thus, we set those five samples as an individual group and conducted a subsequent analysis of variance of the following late PPROM subgroups: five outlying samples with the presence of both MIAC and HCA, the remaining ten samples with the presence of both MIAC and HCA, MIAC alone, HCA alone, or the absence of both MIAC and HCA. We applied the Benjamin-Hochberg correction with the FDR threshold 0.01 in this case.

**Figure 3:**
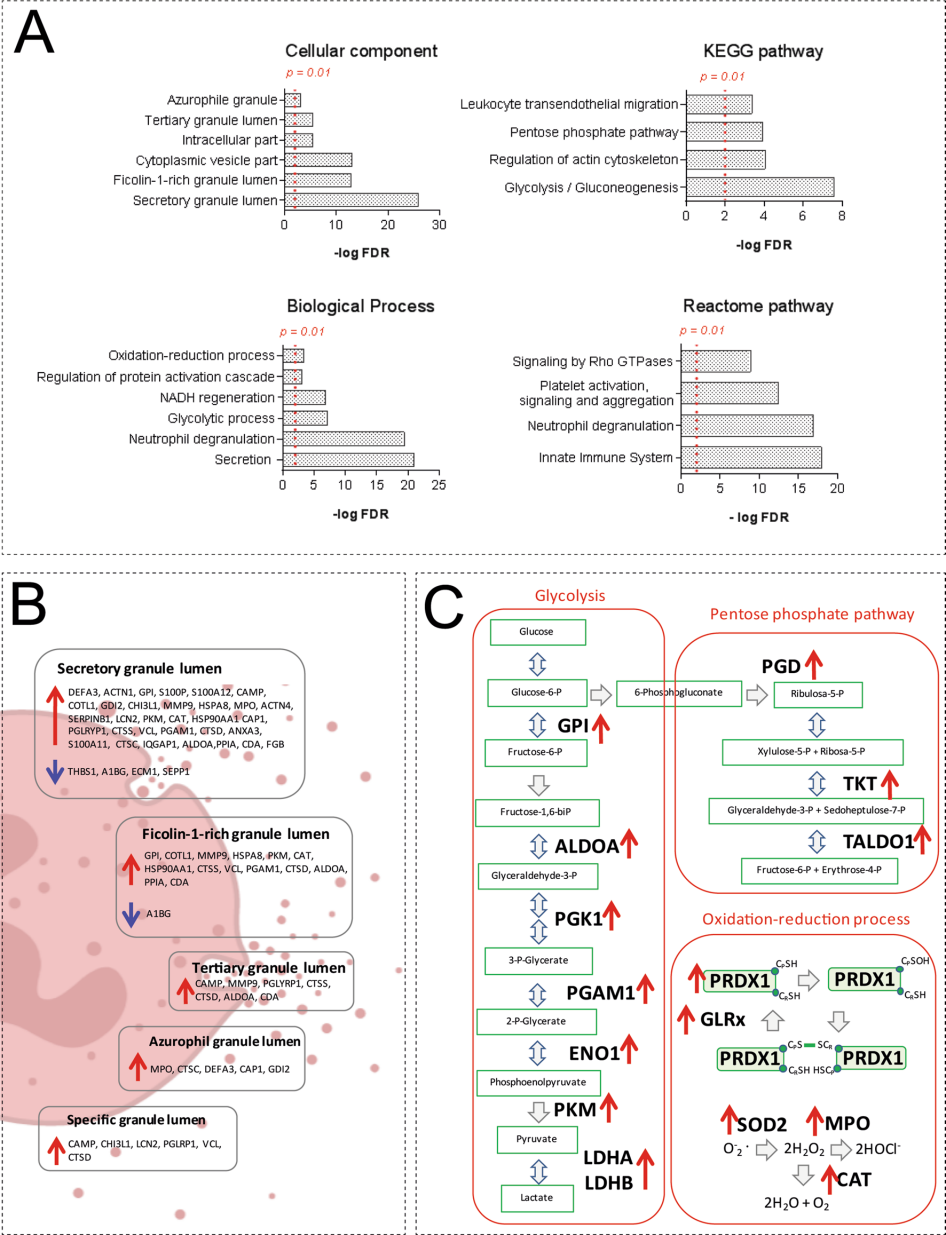
(**A**) Gene ontology analysis of 138 proteins found as significantly changed in the five outlying samples with the presence of both MIAC and HCA. The most over-represented terms of cellular localization, biological process, and pathways are depicted. The FDR *p* value threshold of 0.01 is marked. The gene-ontology terms were retrieved from the STRING enrichment web service using the StringApp in the Cytoscape 3.6.0 (https://cytoscape.org/). (**B**) Change of neutrophil granular proteins. Many of the proteins present in neutrophil granules were found to be significantly up (red arrow) or downregulated (blue arrow) in the five outlying samples with the presence of both MIAC and HCA. (**C**) The upregulation of enzymes participating in glycolysis, the pentose phosphate pathway and redox processes. Upregulation of enzymes participating in the main metabolic neutrophil pathways (glycolysis and pentose phosphate pathway) and killing mechanisms related to neutrophil extracellular traps (redox processes) are depicted (red arrows). The illustration of neutrophil degranulation was created with BioRender.com. Abbreviations: A1BG—alpha-1B-glycoprotein; ACTN1—alpha actinin 1; ACTN4—alpha actinin 4; ALDOA—aldolase; ANXA3—annexin A3; CAMP—cathelicidin; CAP1—adenylyl cyclase-associated protein 1; CAT—catalase; CDA—cytidine deaminase; CHI3L1—chitinase-3-like protein 1; COTL1—coactosin-like protein; CTSC—dipeptidyl peptidase 1; CTSD—cathepsin D; CTSS—cathepsin S; DEFA3—neutrophil defensin 3; ECM1—extracellular matrix protein 1; ENO1—alpha-enolase; FGB—fibrinogen; GDI2—Rab GDP dissociation inhibitor beta; GLRx—glutaredoxin; GPI—glucose-6-phosphate isomerase; HSPA8—heat shock cognate 71 kDa protein; HSP90AA1—heat shock protein HSP 90-alpha; IQGAP1—ras GTPase-activating-like protein IQGAP1; MMP9—matrix metalloproteinase-9; MPO—myeloperoxidase; LCN2—neutrophil gelatinase-associated lipocalin; LDHA—L-lactate dehydrogenase A chain; LDHB—L-lactate dehydrogenase B chain; PGAM1—phosphoglycerate mutase 1; PGD—6-phosphogluconate dehydrogenase; PGK1—phosphoglycerate kinase 1; PGLRYP1—peptidoglycan recognition protein 1; PKM—pyruvate kinase; PPIA—peptidyl-prolyl cis–trans isomerase A; PRDX1—peroxiredoxin 1; S100A11—protein S100A11; S100A12—protein S100A12; S100P—protein S100P; SEPP1—selenoprotein P; SERPINB1—leukocyte elastase inhibitor; SOD2—superoxide dismutase mitochondrial; THBS1—thrombospondin-1; TKT—transketolase; VCL—vinculin.

The subsequent comparison by ANOVA and computed Tukey’s honestly significant difference uncovered that 138 proteins were significantly up- or downregulated in the five outlying samples compared to all other subgroups. In total, 112 proteins were upregulated and 26 proteins were downregulated (Supplementary table). To further characterize these proteins and explain the distinction of the five samples from the remaining samples with the presence of both MIAC and HCA, we conducted gene ontology analysis using the StringApp plugin^25^. All over-represented biological processes, cellular localization, and pathway terms are summed on Supplementary table and the most significant GO terms are depicted in Fig. 3A. Most of the upregulated proteins were localized in neutrophil granules (Fig. 3B). In terms of neutrophil activity, the protein upregulation was associated with platelet activation, glycolysis, the pentose phosphate pathway, and redox processes (Fig. 3C). By contrast, downregulated proteins were annotated as regulatory proteins (Fig. 3A).

### Correlation of the amniotic fluid proteome with the level of interleukin-6, HCA and MIAC

**Figure 4:**
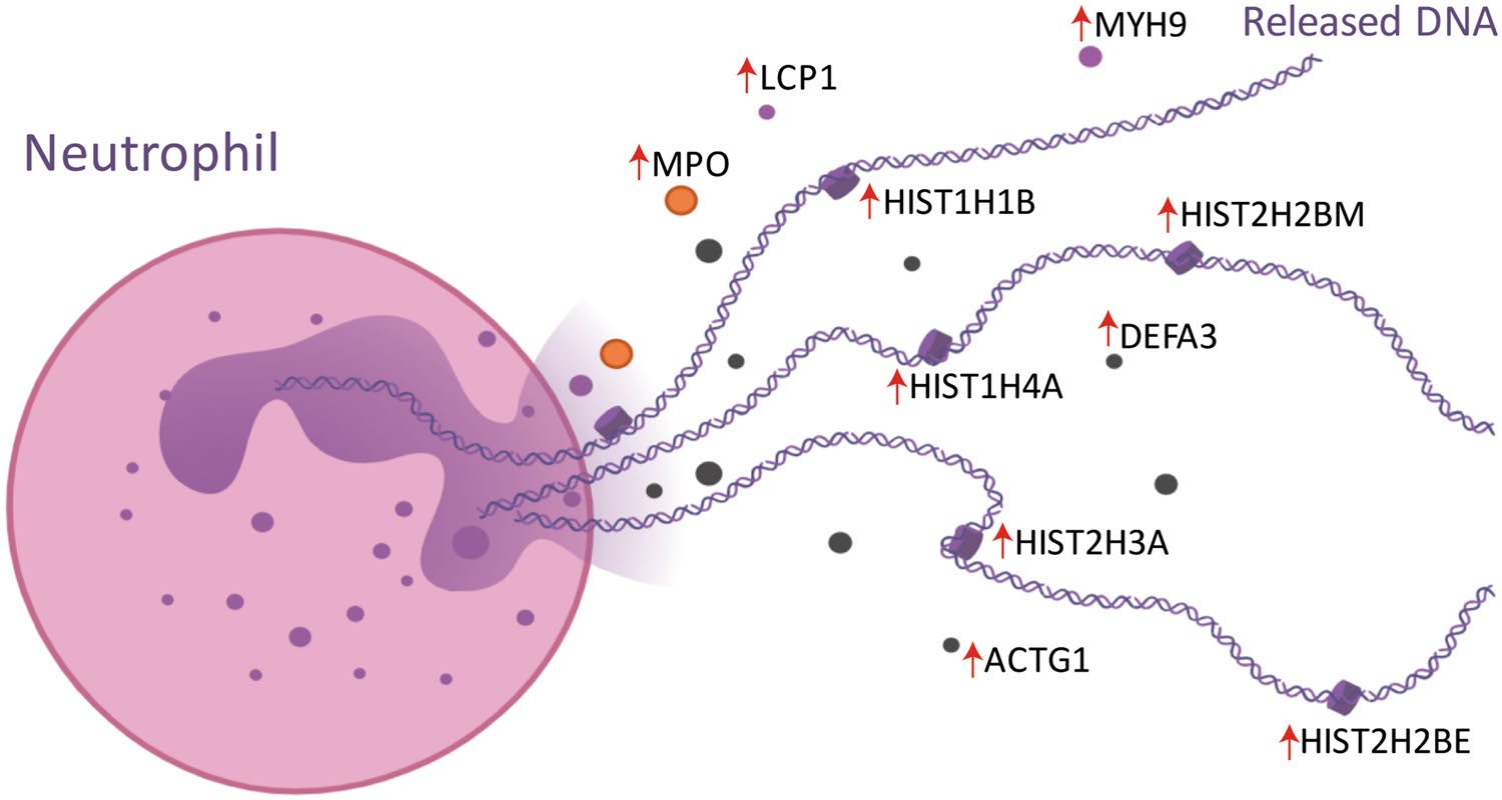
Neutrophil extracellular trap formation. The proteins contributing to the formation of neutrophil extracellular traps and upregulated in the five outlying samples with the presence of both MIAC and HCA are depicted with a red arrow. The illustration of neutrophil extracellular traps was created with BioRender.com. Abbreviations: ACTG1—actin; DEFA3—neutrophil defensin 3; HIST1H1B—histone H1.5; HIST1H4A—histone H4; HIST2H2BM—histone H2B type 1-M; HIST2H3A—histone H3.2; HIST2H2BE—histone H2B type 2-E; LCP1—plastin 2; MPO—myeloperoxidase; MYH9—myosin 9.

**Figure 5:**
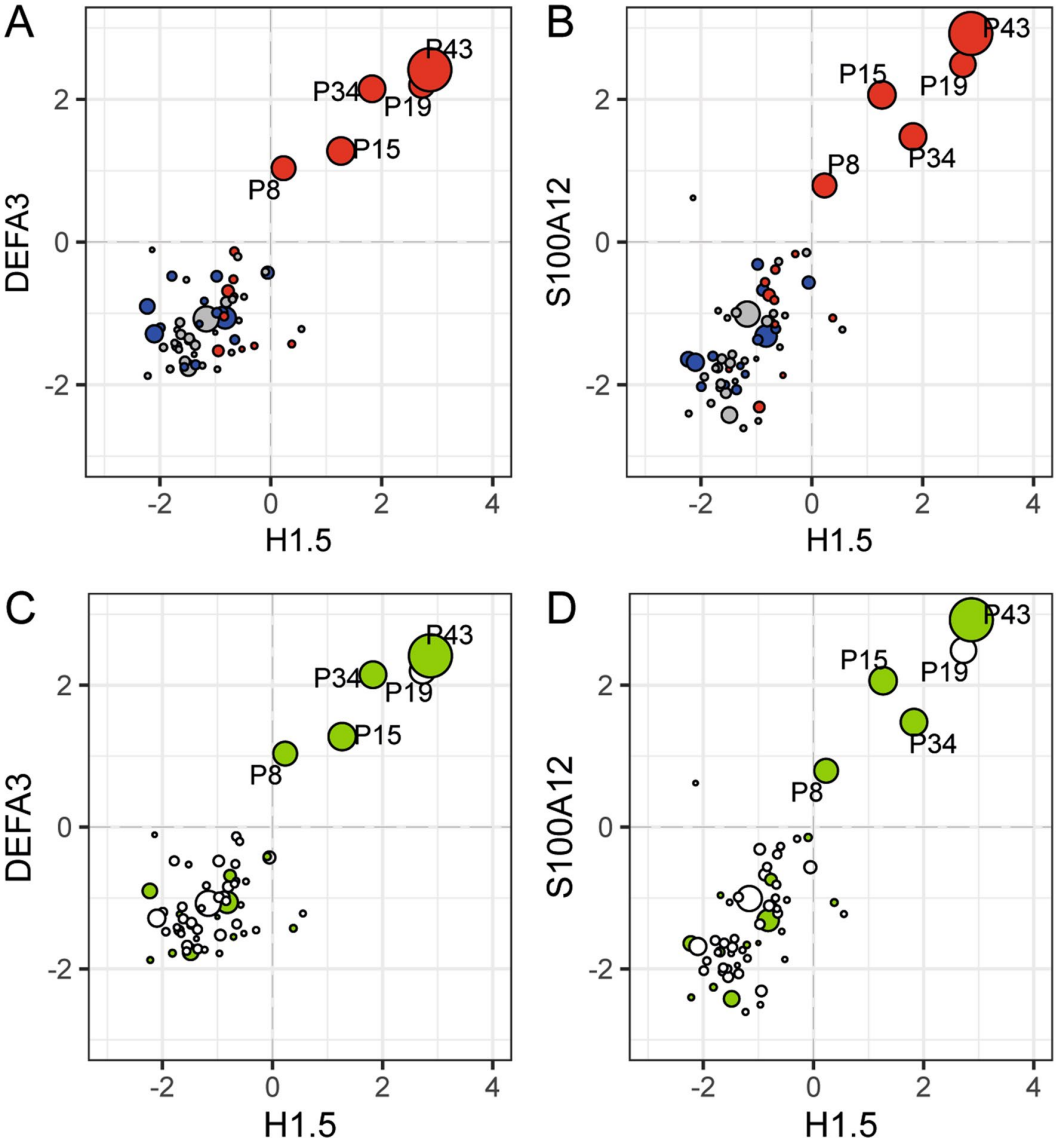
Correlation of the proteomic results with amniotic fluid IL-6 level determined by ELISA, the presence of funisitis, chorioamnionitis and intra-amniotic infection. Amniotic fluid relative quantity of histone H1.5 together with defensin 3 (DEFA3) and protein S100A12 (S100A12) were put into the correlation in the bubble plot. The size of points was set according to the level of IL-6 and the color of points represents the presence of HCA (blue) and funisitis (red) (**A**, **B**). The green-coloured points represent samples with the highest quantity of microbial invasion and/or the presence of *Haemophilus influenzae* and *Sneathia sanquinegens* (**C**, **D**). The five outlying samples were labelled by identification code (P letter and number).

Amniotic fluid IL-6 is currently the most relevant and discussed protein for evidence of intra-amniotic inflammation in PPROM^29^. Accordingly, we determined also the concentration of IL-6 in our samples and examined the correlation of IL-6 with the protein upregulation discovered through TMT 10plex LC–MS/MS analysis. We sought to assess whether proteome differences in the presence of MIAC and/or HCA correspond with the level of IL-6 in amniotic fluid. To this end, we constructed bubble plots as a correlation of H1.5 level with that of two other proteins, neutrophil defensin 3 (DEFA3) and protein S100A12. The size of bubbles are set according to the concentration of IL-6 and the color of bubbles represents the presence or absence of HCA and funisitis, the most severe form of HCA characterized by the presence of neutrophils in the umbilical cord (Fig. 5A,B). Histone H1.5, DEFA3, and S100A12 were found as highly upregulated proteins in the five outlying samples with the presence of both MIAC and HCA (Supplementary table). Moreover, DEFA3 and S100A12 were classified as proteins enriched in the antimicrobial immune response, neutrophil degranulation, and cell secretion (Fig. 3B). DNA-associated histones are frequently discussed as forming neutrophil extracellular traps upon infectious stimuli^30^ (Fig. 4).

Amniotic fluid levels of H1.5, DEFA3 and S100A12 showed substantial enhancement in the case of the five samples with the presence of both MIAC and HCA and also positive correlation with the highest levels of IL-6 (Fig. 5). Together, funisitis was confirmed in these five samples (Fig. 5A,B). These five samples were also found to have a high microbial load of *Ureaplasma* spp. and *M. hominis* DNA (threshold cycle values < 22, responding quantity more than 10^5^ copies/mL) or the presence of *Haemophilus influenzae* and *Sneathia sanquinegens* was confirmed (Fig. 5C,D and Table 2).

**Table 2:** List of pathogens in the five samples from the subgroup with the presence of both MIAC and HCA.

Sample ID	U_Ct	Mh_Ct	Bacteria	IL-6 (pg/mL)	Histopathology
P8	16.02		*Ureaplasma* spp.	13822.33	Funisitis
P15			*Haemophilus influenzae*	18812.52	Funisitis
P19	23.2	23.9	*Ureaplasma* spp., *Mycoplasma hominis*	15857.05	Funisitis
P34	26.3		*Sneathia sanquinegens*, *Ureaplasma* spp.	17675.76	Funisitis
P43	21.8		*Ureaplasma* spp.	49426.60	Funisitis

The list includes the name of pathogens, diagnosis of histopathology and amniotic fluid IL-6 concentration in the five outlying samples, where both MIAC and HCA conditions were confirmed. Only these five samples were associated with proteome alterations. The five outlying samples were described by identification code (P letter and number).

Abbreviations: U—*Ureaplasma* spp., Mh—*Mycoplasma hominis*, Ct—Cycle of threshold.

### Validation of amniotic fluid histone H1.5

**Figure 6:**
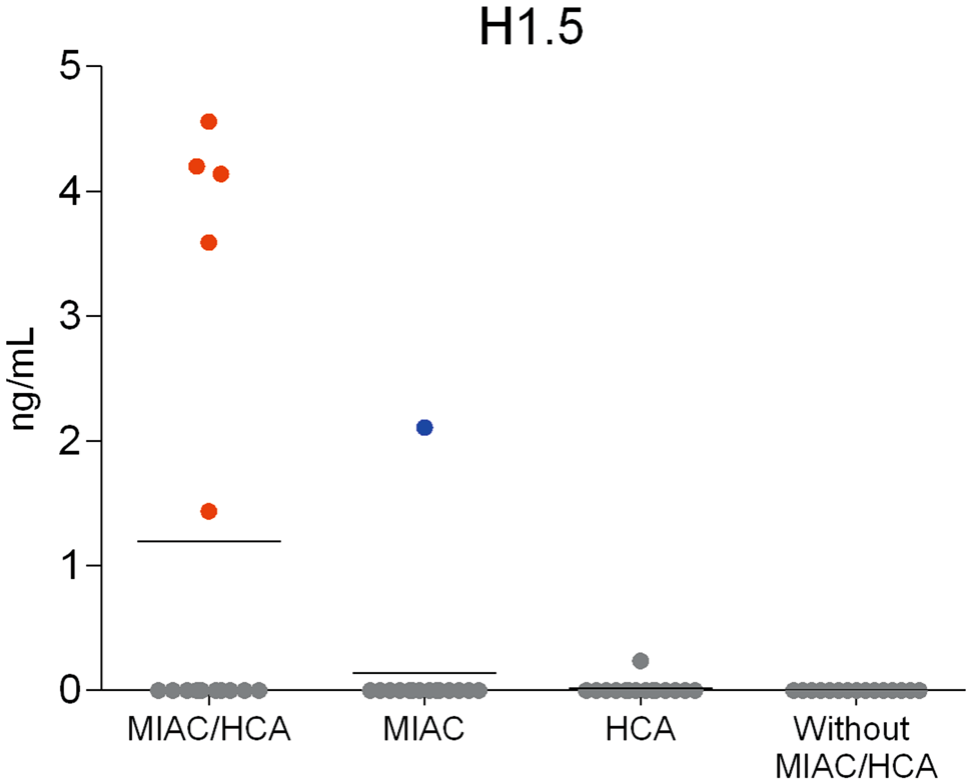
Amniotic fluid level of histone H1.5 determined by ELISA. The concentration of H1.5 was zero in almost all samples regardless of the presence or absence of MIAC and/or HCA, except for the five outlying samples from the subgroup with the presence of both MIAC and HCA, and one sample with the MIAC alone. The level of H1.5 of the five outlying samples from the subgroup with the presence of both MIAC and HCA are highlighted as red points. The H1.5 level of 2.11 ng/mL in the sample with the presence of MIAC alone is highlighted in blue.

As aforementioned, amniotic fluid histone H1.5 level was found to be significantly higher in the five samples from the subgroup with the presence of MIAC and HCA (Supplementary table). Histone H1.5 is an important intracellular protein related to excessive inflammatory response^31^. Thus, we determined the amniotic fluid level of histone H1.5 in the subgroups with the presence or absence of MIAC and/or HCA using ELISA. All but six samples had the level of H1.5 on concentration below the limit of detection of the ELISA kit (Fig. 6). The H1.5 level of the five outlying samples from the subgroup with both MIAC and HCA was in the range from 1.44 to 4.56 ng/mL. The sixth sample with H1.5 level of 2.11 ng/mL belonged to the group with MIAC alone but the IL-6 level of this sample was more than 15,000 pg/mL despite the absence of chorioamnionitis or funisitis (Fig. 6).

## Discussion

In this study, we utilized a quantitative proteomic approach based on isobaric labeling of peptides to reveal proteome differences related to the MIAC and HCA in women with late PPROM. The principal findings of this study are: (1) The levels of amniotic fluid proteins in the subgroup with both MIAC and HCA were surprisingly uniform compared to the other late PPROM subgroups where both conditions weren’t confirmed. We found only five cases of late PPROM women with a substantial protein dysregulation related to both MIAC and HCA. We speculate that particularly these women can profit from an active management beyond the 34th week of gestation. (2) Proteome change was associated with a high concentration of IL-6 in amniotic fluid. (3) The positive correlation of proteomic findings with IL-6 concentration identified five women with strong intra-amniotic inflammation in late PPROM.

In our previous reports, LC–MS/MS enabled us to reveal changes in amniotic fluid proteome associated with the presence of MIAC and HCA in PPROM from 24 to 36th week of gestation^17,20,32^. However, to the best of our knowledge, a global proteomic study focused on PPROM beyond the 34th week has not been carried out yet. In TMT 10plex-based quantification, peptides of each sample are tagged with one different isotopic variant from a set of ten mass tags^16,33^. However, since the number of patients in our late PPROM cohort exceeded the number of TMT channels, we designed seven multiplexes and analyzed them in different LC–MS/MS runs (Fig. 1A). Normalization of mass tag information to the GIS removed the eventual intensity fluctuation during LC–MS/MS analysis of a particular multiplex, e.g. due to instability of electrospray (Fig. 1B).

Protein abundance may vary by orders of magnitude in complex samples. To make this variance similar across the global amniotic fluid proteome, the data were log_2_ transformed and normalized to the median^34^. Also, a high number of missing values may debase mass tag-based quantification and provide incorrect information about protein levels^35^. Perseus software replaces the missing values by random numbers that are estimated from normal distributions^23^, but that would mean working with a dataset containing artificial values. Hence, we rather decided to exclude proteins with a high number of missing values in technical replicates of analyzed samples (Fig. 1B).

This study was inspired by published results which indicated that a change of amniotic fluid proteome is associated with the presence of both MIAC and HCA in PPROM^20,32^. The authors applied a CysTRAQ approach, consisting of iTRAQ labeling to quantify selectively the fraction of peptides with cysteine residue and the fraction of non-cysteinyl peptides and found more than 80 amniotic fluid proteins significantly dysregulated in the presence of MIAC and HCA in PPROM pregnancies. Histone H4 together with other antimicrobial proteins (cathelicidin, neutrophil defensin 1, myeloperoxidase, etc.) and proteins integrated in neutrophil extracellular traps (myosin-9, plastin-2, matrix metalloproteinase-9, etc.) were shown to be considerably upregulated in the presence of both MIAC and HCA. Besides the CysTRAQ article, recent papers have discussed the elevation of IL-6 in the presence of both MIAC and HCA in cases of both PPROM and late PPROM^13,36^. The authors also concluded that the presence of both MIAC and HCA and a higher level of IL-6 were related to early-onset sepsis and neonatal morbidity.

In the CysTRAQ study, a pooled sample strategy was used. The pooling of samples is a time-efficient strategy compensating for limited amounts of the samples or high biological variation^37^. However, protein inflammatory response to MIAC and HCA is not a homogeneous phenomenon and a pooling strategy may disguise the variance of inflammatory protein concentration in individual samples. Keeping in mind the heterogeneity of MIAC and HCA, we required information about levels of proteins in each individual sample of the patient cohort. Moreover, we included also the subgroups with MIAC alone and HCA alone. Histological chorioamnionitis itself may evoke the pro-inflammatory protein response, but only the presence of both MIAC and HCA is apparently related to a higher number of cases of early-onset sepsis and fetal inflammatory response syndrome^13,38^. Hence, it is desirable to distinguish women with both MIAC and HCA from those with HCA alone.

Analysis of data achieved through TMT 10plex LC–MS/MS and correlation of these data with the IL-6 concentration in amniotic fluid, diagnosed funisitis, and MIAC revealed the substantial disparity in the subgroup with the presence of both MIAC and HCA. Dysregulation of amniotic fluid proteins occurred only in five cases in the subgroup with the presence of both MIAC and HCA whereas no significant distinction of protein level was observed in the remaining 55 entities regardless of MIAC and/or HCA (Fig. 2). These five samples were also affected by a high DNA load of *Ureaplasma* spp. and/or *M. hominis* and/or the presence of *Haemophilus influenzae* and *Sneathia sanquinegens* (Fig. 5C,D, Table 2)*.* Nevertheless*,* some of the samples from women with MIAC alone with a low level of protein determined by LC–MS/MS as well as a low level of IL-6 were also affected by a high load of bacterial DNA (Fig. 5C,D). This confirmed previous findings that MIAC can occur as isolated bacterial colonization without any inflammation-associated change of proteome^2^. These five “outliers” were also diagnosed as pregnancies with funisitis (Fig. 5A,B). Funisitis is defined as the presence of neutrophils in the wall of umbilical vessels and/or Wharton’s jelly^39^. It seems that these five cases of the subgroup with both MIAC and HCA are the most affected cases with potential severe fetal inflammatory involvement.

A total of 138 proteins were significantly up- or downregulated in the five samples of the group with the presence of both MIAC and HCA compared to the other samples from women with late PPROM (Supplementary table). Substantial portion of upregulated proteins was annotated as secreted proteins present in neutrophil granules, proteins participating in glycolysis and the pentose phosphate pathway, in the regulation of cytoskeletal actin, and redox process (Fig. 3). The downregulated proteins were classified as regulators of acute inflammatory response. Neutrophil degranulation and secretion is host defense mechanism against potential intra-amniotic infection^40^. Upon cell activation, antimicrobial proteins such as myeloperoxidase (MPO), DEFA3, cathelicidin are released from neutrophil granules^20,41^. The lumen of neutrophil granules was the most over-represented cellular component in our GO analysis (Fig. 3A).

Besides the granular proteins mentioned above, we found other 34 proteins present in neutrophil granules and most of them were highly upregulated in the five discussed samples (Fig. 3B). Upregulated calcium-binding S100 proteins are small secreted proteins crucial for neutrophil migration and they may act as alarmins in the pathology of funisitis and early neonatal sepsis^42^. In addition, protein S100-A12 has a function as a ligand for the receptor for advanced glycation end‐products (RAGE)^43^. The ligand-RAGE interplay is a key mechanism of reactive oxygen species (ROS) generation and oxidative burst^44^. Other target molecule of S100 proteins is glyceraldehyde-3-phosphate dehydrogenase (GAPDH), a key enzyme of glycolysis^45^. Glycolysis and the pentose phosphate pathway (PPP) are the key metabolic pathways in neutrophils and we found both of them as significant pathways in GO enrichment (Fig. 3A). We observed an expressive upregulation of enzymes participating in glycolysis and pyruvate generation in the five samples of the subgroup with the presence of both MIAC and HCA (Fig. 3C).

Besides energy generation, glycolysis and PPP directly affect also the formation of ROS by providing NADPH. A total of 19 proteins related to the redox process were upregulated in the five samples. We found upregulation of enzymes important for formation and elimination of ROS, including MPO, mitochondrial superoxide dismutase, catalase, glutaredoxin-1, peroxiredoxin-1, etc. (Supplementary table, Fig. 3C). Glycolysis, PPP and MPO- induced ROS may directly affect releasing of neutrophil extracellular traps (NETs)^46–48^. The presence of NETs in intra-amniotic infection and inflammation has been already established^49^. Unambiguous confirmation of NETs in amniotic fluid for instance using immunofluorescence microscopy^50^ was beyond the scope of this study. Nevertheless, we found alterations in the redox homeostasis affecting the neutrophil activity towards to NETs formation as well as the substantial upregulation of proteins being a part of NETs defense mechanisms such as MPO, DEFA3, MMP9 and histone isoforms representing strong indicia of the NETs presence (Fig. 4)^51^.

Histones are frequently discussed as key part of NETs and potential biomarkers of MIAC and HCA in PPROM^20,32,49^. In the five outlying samples from the subgroup with both MIAC and HCA, histones H1.5, H2B, H4 and H3.2 were highly upregulated. The level of H1.5 in amniotic fluid determined by ELISA also showed a significant increase in the five outlying samples with the presence of both MIAC and HCA compared to the other 55 samples (Fig. 6). Beyond the direct antimicrobial function, extracellular histones in released NETs bind platelets and induce platelet aggregation^52^. NETs have been even signed as a link between inflammation and thrombosis^53^. We found twenty up- or downregulated proteins related to platelet activation, signaling and aggregation (Fig. 3A, Supplementary table). From those, cofilin-1 and actinins were the most upregulated. Cofilin-1 is an actin-binding protein responsible for actin re-modelling preceding platelet aggregation^54,55^. On the other hand, another platelet protein thrombospondin-1 was downregulated in the five outlying samples with the presence of both MIAC and HCA compared to the other 55 samples. Thrombospondin-1 inhibits the activity of neutrophil elastase and cathepsin G and mitigates the inflammatory response^56^. We suggest that downregulation of thrombospondin-1 may promote the activity of neutrophil proteases as a consequence of excessive intra-amniotic inflammation in the specific subgroup with the presence of both MIAC and HCA in late PPROM.

## Conclusion

In this study, we analyzed a unique collection of late PPROM amniotic fluid samples without a significant difference in gestational age at PPROM, amniocentesis, and delivery. We revealed the dysregulation of 138 proteins along with the high level of amniotic fluid IL-6, the presence of funisitis and MIAC. This study was initially aimed at discovery of protein changes associated with MIAC and HCA. We had presumed that the presence of neutrophils in the placenta, umbilical cord, and fetal membranes would be reflected by the change in the amniotic fluid proteome. However, we only detected five samples in the subgroup with the presence of both MIAC and HCA with substantial change in proteome composition. Consequently, the original stratification of the late PPROM cohort based on the status of HCA turned out to be a limitation of this study because the number of MIAC/HCA women with a strong proteome response was very low. However, these women can particularly profit from active management compared to other women with late PPROM. With respect to the achieved results, we recommend diagnosing the intra-amniotic inflammation rather based on the level of amniotic fluid IL-6 than as the diagnosed HCA. Nevertheless, the threshold values of intra-amniotic inflammation in PPROM vary from 2.6 to 11.3 ng/mL in previous publications^57^, and the conclusive cut-off value has not been established. Future research should thus unambiguously define the concentration of IL-6 representing the threshold for the confirmation of intra-amniotic inflammation.

## Supplementary information

Supplementary information.

Supplementary table.

## Data Availability

The mass spectrometry data together with the MaxQuant output files have been deposited in the ProteomeXchange Consortium via the PRIDE partner^58^ repository with the dataset identifier PXD020329.
