# Supplementary material for: Comprehensive proteomic investigation of infectious and inflammatory changes in late preterm prelabour rupture of membranes
Source: Sci Rep. 2020 Oct 19;10:17696. doi: 10.1038/s41598-020-74756-9 (PMC7573586; doi:10.1038/s41598-020-74756-9)
Supplement: Supplementary file 2 — Supplementary table. [file 41598_2020_74756_MOESM2_ESM.pdf]

Supplementary Document

to the Original Research Article

Comprehensive proteomic investigation of infectious and inflammatory changes in late preterm prelabour rupture of membranes

Marie Vajrychová[1*], Jaroslav Stráník[2], Kristýna Pimková[1a], Malin Barman[3], Rudolf Kukla[4], Petra Zedníková[1,5b], Radka Bolehovská[4], Lenka Plíšková[6], Helena Hornychová[7], Ctirad Andrýs[8], Vojtěch Tambor[1], Juraj Lenčo[1,9], Bo Jacobsson[10,11], Marian Kacerovský[1,2*]

[1]Biomedical Research Center, University Hospital Hradec Kralove, Hradec Kralove, Czech Republic

[2]Department of Obstetrics and Gynecology, University Hospital Hradec Kralove, Charles University, Faculty of Medicine in Hradec Kralove, Hradec Kralove, Czech Republic

[3]Food and Nutrition Science, Department of Biology and Biological Engineering, Chalmers University of Technology, Gothenburg, Sweden

[4]Institute of Clinical Microbiology, University Hospital Hradec Kralove, Hradec Kralove, Czech Republic

[5]Department of Biological and Biochemical Sciences, Faculty of Chemical Technology, University of Pardubice, Pardubice, Czech Republic

[6]Institute of Clinical Biochemistry and Diagnostics, University Hospital Hradec Kralove, Hradec Kralove, Czech Republic

[7]Fingerland's Department of Pathology, University Hospital in Hradec Kralove, Charles University, Faculty of Medicine Hradec Kralove, Hradec Kralove, Czech Republic

[8]Institute of Clinical Immunology and Allergology, University Hospital Hradec Kralove, Hradec Kralove, Czech Republic

[9]Department of Analytical Chemistry, Faculty of Pharmacy in Hradec Kralove, Charles University in Prague, Hradec Kralove, Czech Republic

[10]Department of Obstetrics and Gynecology, Institute of Clinical Science, Sahlgrenska Academy, University of Gothenburg, Gothenburg, Sweden

[11]Department of Genetics and Bioinformatics, Domain of Health Data and Digitalization, Institute of Public Health, Oslo, Norway

Present address:

[a]BIOCEV, First Faculty of Medicine, Charles University, Prague, Czech Republic

[b]Department of Metabolomics, Institute of Physiology, Czech Academy of Sciences, Prague, Czech Republic

***e-mail:** marie.vajrychova@fnhk.cz; marian.kacerovsky@fnhk.cz

Appendix S1: Methods

Affinity depletion of ballast amniotic fluid proteins

Amniotic fluid ballast proteins were depleted using Multiple Affinity Removal System (MARS) Hu-14 (Agilent Technologies, Santa Clara, CA, USA). Equal amounts of protein (500 μg) were taken from all samples and amniotic fluid proteins were transferred to a MARS buffer using Amicon centrifugal filters with a 3 kDa cut-off membrane (Millipore, Bedford, MA, USA). The retenates were adjusted to 200 μL with the MARS buffer A and depleted using high-performance liquid chromatography system Alliance 2695 (Waters, Milford, MA, USA). After that, all samples were passed through 3 kDa cut-off Amicon filters to exchange the MARS buffer A for water and to attain the protein amount of 30 μg in 140 μL.

Peptide cleaning

Multiplexes were completely evaporated, redissolved in 100 μL of 100 mM TEAB and incubated at 30°C for 30 minutes. Next, the multiplex samples were acidified with trifluoroacetic acid (TFA) (Thermo Scientific, Rockford, IL, USA) at a final concentration of 1%. Sodium deoxycholate was extracted into water-saturated ethyl acetate (EA). Multiplex samples were shaken with 200 μL of EA followed by centrifugation at 10,000 x g for two minutes. The organic phase was discarded, and the whole procedure was repeated five times. Between EA additions, the multiplex samples were acidified repeatedly as described above. Remaining EA traces in the aqueous phase were evaporated, and 0.1% TFA in 5% acetonitrile (AcN) (Honeywell, Charlotte, NC, USA) was added into a final volume of 1 mL. Multiplexed samples were centrifuged at 14,500 rpm for 10 minutes. After that, the supernatants were loaded onto the C18 solid phase of Empore C18-SD SPE cartridges (3M, St. Paul, MN, USA), prewashed with 1 mL of methanol and equilibrated with 0.1% TFA in 5% AcN. Then, the C18 phase was washed twice with 1 mL of 0.1% TFA in 5% AcN, and trapped peptides were released using 300 μL of 0.05% TFA in 50% AcN. Finally, all the multiplex samples were vacuum-dried.
